# Immune Response of A Novel ATR-AP205-001 Conjugate Anti-hypertensive Vaccine

**DOI:** 10.1038/s41598-017-12996-y

**Published:** 2017-10-03

**Authors:** Xiajun Hu, Yihuan Deng, Xiao Chen, Yanzhao Zhou, Hongrong Zhang, Hailang Wu, Shijun Yang, Fen Chen, Zihua Zhou, Min Wang, Zhihua Qiu, Yuhua Liao

**Affiliations:** 1 Department of Cardiology, Union Hospital, Tongji Medical College, Huazhong University of Science and Technology, Wuhan, 430022 China; 2 Institute of Cardiology, Union Hospital, Tongji Medical College, Huazhong University of Science and Technology, Wuhan, 430022 China; 3 Key Lab of Molecular Biological Targeted Therapies of the Ministry of Education, Union Hospital, Tongji Medical College, Huazhong University of Science and Technology, Wuhan, 430022 China

## Abstract

We developed a virus-like particle (VLP)-based therapeutic vaccine against angiotensin II receptor type 1, ATR-AP205-001, which could significantly reduce the blood pressure and protect target organs of hypertensive animals. In this study, we focused on the immunological effect and safety of the VLP-based vaccine. By comparing to the depolymerized dimeric vaccine ATR-Dimer-001, we found that ATR-AP205-001 reached subcapsular sinus of lymph node shortly after administration, followed by accumulation on follicle dendritic cells via follicle B cell transportation, while ATR-Dimer-001 vaccine showed no association with FDCs. ATR-AP205-001 vaccine strongly activated dendritic cells, which promoted T cells differentiation to follicular helper T cells. ATR-AP205-001 vaccine induced powerful germinal center reaction, which was translated to a boost of specific antibody production and long-lasting B cell memory, far superior to ATR-Dimer-001 vaccine. Moreover, neither cytotoxic T cells, nor Th1/Th17 cell-mediated inflammation was observed in ATR-AP205-001 vaccine, similar to ATR-Dimer-001 vaccine. We concluded that ATR-AP205-001 vaccine quickly induced potent humoral immunity through collaboration of B cells, follicular dendritic cells and follicular helper T cells, providing an effective and safe intervention for hypertension in the future clinical application.

## Introduction

Primary hypertension is a chronic disease with high morbidity and mortality. The rate of controlled blood pressure and the treatment compliance are far from satisfactory, worldwide^1^. One of the most important pathogenesis of hypertension is over-activation of renin-angiotensin system (RAS). Classic RAS is composed by an axis of renin-angiotensin converting enzyme (ACE)-angiotensin II (Ang II)-angiotensin II receptor type 1 (AT1R)^2^. Ang II is one of the strongest vasoconstrictor agent. AT1R, the major receptor of Ang II, mediated pressor effect and target organs damage induced by Ang II. Each part of the axis can be the target of anti-hypertension. Renin inhibitors, ACE inhibitors (ACEIs) and AT1R blockers (ARBs) are the main therapeutic drugs in clinical practice. Nevertheless, all the drugs need to be taken daily, consistently, and even permanently, which undoubtedly reduces patients compliance and increases the economic burden.

Therapeutic vaccine is a new approach for neoplastic diseases, cardiovascular and cerebrovascular diseases^3^. Our team invented a virus-like particle (VLP)-based anti-hypertensive vaccine against AT1R, which could significantly lower the blood pressure and protect target organs of hypertensive animals^4^, even ameliorate atherosclerosis^5^ and nephropathy^6^ in animal models. AT1R-VLP vaccine is injected into the animals every two to four weeks. The half life (14.4 days) of productive antibody is much longer than existing anti-hypertensive chemical drugs^4^, which indicates AT1R-VLP vaccine reduces blood pressure more steadily. In addition, obvious RAS feedback activation which makes the effect of ARBs somewhat self-limiting was not found in vaccinated animals^6,7^. All these advantages support that AT1R-VLP vaccine is a novel and promising intervention to hypertension.

ATR-AP205-001 vaccine is produced by chemical conjugation of ATR001 to AP205 VLP carrier, similar to our previous AT1R-VLP vaccine^4^. ATR001 is a linear B cell epitope composed of 7 amino acids (Ala-Phe-His-Tyr-Glu-Ser-Gln), derived from the extracellular loop 2 of human AT1R. As a small self-antigen, ATR001 alone is hard to induce immune response in normal condition because of non-recognition or tolerance. VLP is one of the self-assembled nanoparticles with a diameter of 25–100 nm, composed of repetitive coat protein, while lacking the virus genomes. Even though macromolecules as VLP cannot diffuse randomly to the follicles like soluble antigens^8^, particle structure and highly repetitive epitopes give VLP ideal antigenicity than subunit and recombinant protein immunogens in vaccine design^9,10^. AP205 VLP could present antigens in regular and iterative array which is of benefit to antigen processing by antigen presenting cells (APCs). VLP is easy to bind to B cells through BCRs because of high repetitive epitopes presented on the surface. Some researchers even think VLP is T cell-independent antigens because of their high affinity and activation ability to B cells^11^. Since the discovery of VLP, it has been widely used in vaccine development. Now several recombinant vaccines had been commercialized^12,13^, and more experimental vaccines are in research and development^14,15^. Strong humoral immunity is the most important concern of our AT1R vaccine. Conjugatio﻿n of ATR001 with AP205 VLP carrier (designated ATR-AP205-001) should be﻿ an ﻿entirely feasible strategy to overcome se﻿lf-tolerance of ATR001 and achieve ideal humoral immune response.﻿

To date, no specialized research was carried out to illuminate the immune response and safety mechanism of VLP-peptide vaccine. At the moment, preclinical study of AT1R-VLP vaccine is being in progress. To accelerate the clinical transformation of AT1R-VLP vaccine, ATR-AP205-001 and the depolymerized protein vaccine ATR-Dimer-001 were produced to explore the immune response characteristics. The way of uptake and trafficking of vaccines into the lymphatic follicles was traced *in vivo*. And how the VLP-based vaccine activated immune system and induced efficient humoral immune response was investigated.

## Results

### ATR-AP205-001 vaccine reduced blood pressure and protect target organ in Ang II-induced hypertensive mice

**Figure 1:**
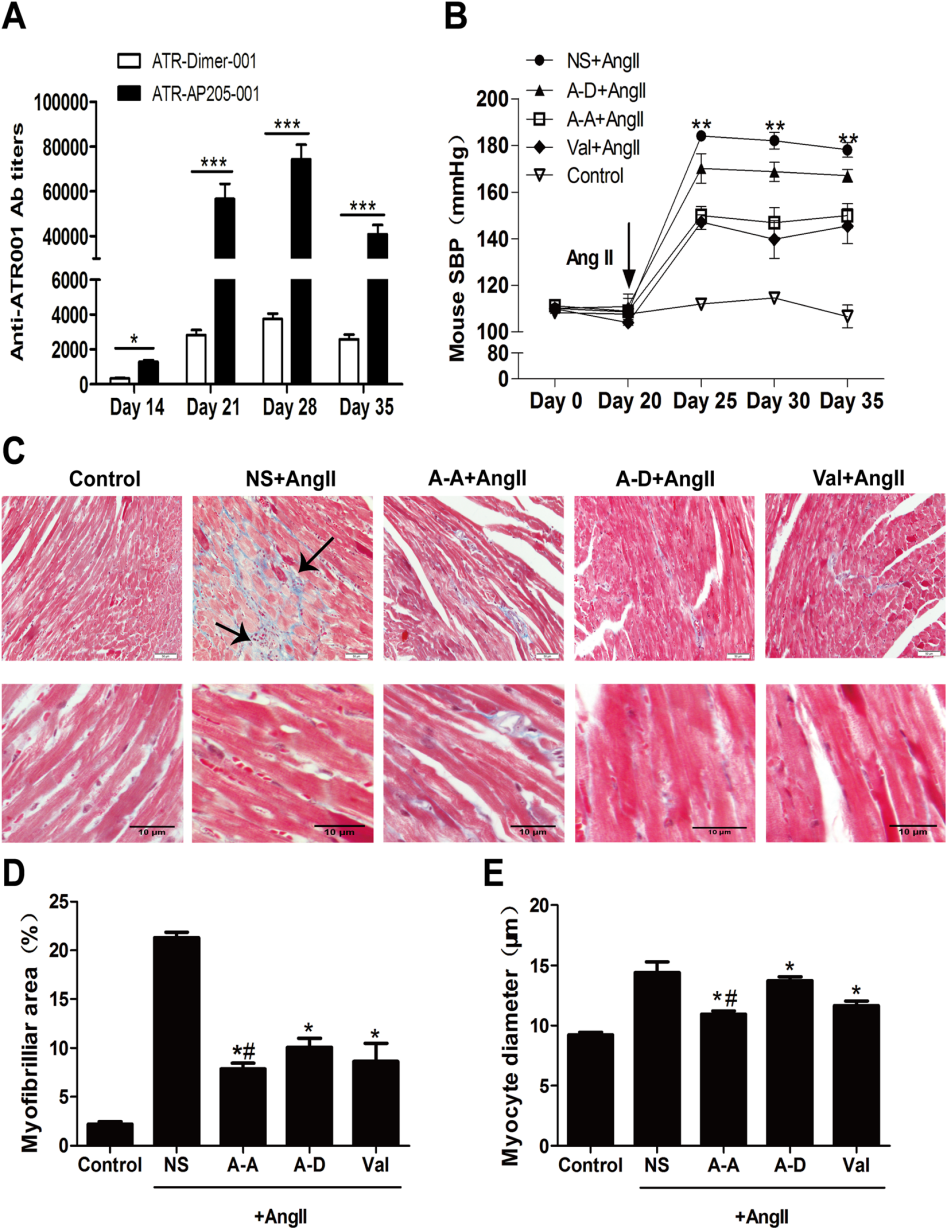
ATR-AP205-001 efficiently reduced blood pressure and target organ damages of Ang II induced hypertensive mice. (**A**) Anti-ATR001 antibody titers were measured by ELISA on days 14, 21, 28 and 35 after ATR-AP205-001 and ATR-Dimer-001 vaccination. n = 8 per group. *P < 0.05, ***P < 0.001 vs. ATR-Dimer-001. (**B**) Blood pressure of mice was tested by tail-cuff method on days 0, 20, 25, 30 and 35. **P < 0.01 vs. NS + Ang II group. (**C**) Masson trichrome staining identified fibrosis (marked by black arrows) of heart tissue in each group. Cardiomyocyte diameter was identified by the length perpendicular to the long axis of the cell (n = 20 myocytes per group). Scale bars, 50 μm (upper) and 10 μm (lower). (**D**) The ratio of fibrotic area to total heart tissue and (**E**) myocytes diameter tested in ImageJ. *P < 0.05 vs. NS + Ang II group, ^#^P < 0.01 vs. ATR-Dimer-001 group. A-A: ATR-AP205-001, A-D: ATR-Dimer-001, Val: valsartan, and NS: natural saline in this Figure. Data are expressed as mean ± SEM.

We used Ang II-induced hypertensive mice to evaluate the efficacy of vaccination. BALB/c mice were vaccinated on days 0 and 14. ELISA testing confirmed the anti-ATR001 antibody titers in ATR-AP205-001 group and ATR-Dimer-001 group (Fig. 1A). Mice were infused with Ang II osmotic pumps (1.4 mg/kg/day) on day 21. Both ATR-AP205-001 vaccine and valsartan treatment obviously reduced systolic blood pressure (SBP), while ATR-Dimer-001 vaccine showed minor effect (Fig. 1B). Compared with the saline group (21.30 ± 0.54%), myocardium fibrosis was significantly attenuated in ATR-AP205-001 vaccine group (7.85 ± 0.60%, P < 0.05) and ATR-Dimer-001 vaccine group (10.07 ± 0.95%, P < 0.05) (Fig. 1C,D). ATR-AP205-001 vaccine also performed better in preventing excess myocardial fiber hypertrophy compared to ATR-Dimer-001 vaccine group (fiber diameter: 10.96 ± 0.26μm vs. 13.75 ± 0.30 μm, P < 0.05) (Fig. 1C,E). Furthermore, no inflammatory damage was observed in heart and kidney of normal BALB/c mice after 5 times immunization (see Supplementary Fig. S1).

### ATR-AP205-001 vaccine rapidly accumulated and deposited in FDC area of lymph node

**Figure 2:**
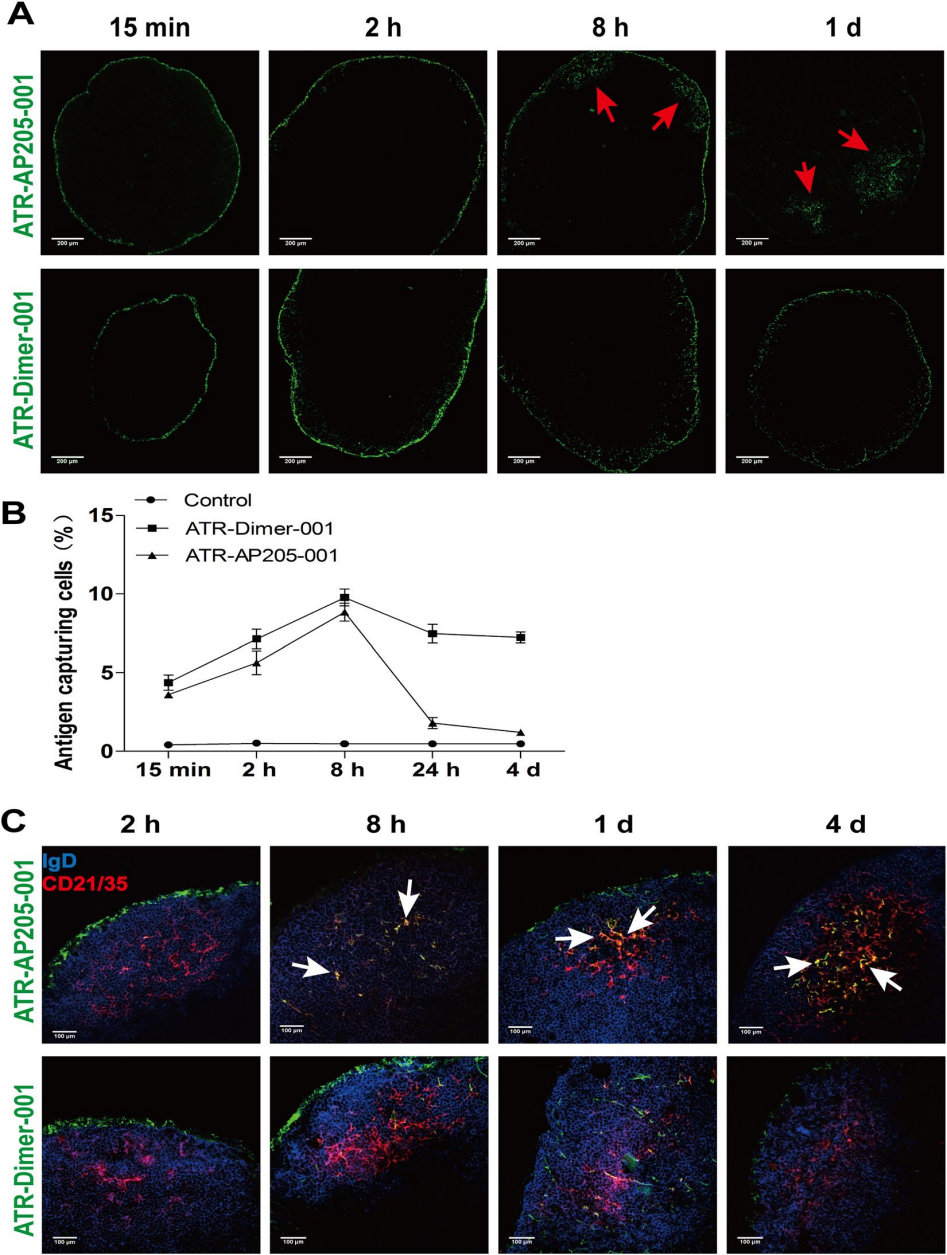
ATR-AP205-001 was drained to popliteal lymph node and deposited in follicle dendritic cell (FDC) area. Mice popliteal lymph nodes were acquired at 15 mins, 2 hrs, 8 hrs, 24 hrs and 4 days after footpad injection. (**A**) Distribution of ATR-AP205-001 (upper, green) and ATR-Dimer-001 (lower, green) in popliteal lymph node. Red arrows figure out the accumulation of antigens inside the lymph node. Scale bars, 200 μm. (**B**) Proportion of cells participating in antigen-capturing. (**C**) Co-localization of ATR-AP205-001 (upper, green) and ATR-Dimer-001 (lower, green) with IgD^+^ follicle B cells (blue) and CD21/35^+^ FDCs (red). The antigens deposited in FDC area were annotated by white arrow. Scale bars, 100 μm. n = 6 per group. Data are expressed as mean ± SEM.

To track the appearance and deposition of vaccine in lymphoid organs, the FITC-labeled ATR-AP205-001 and ATR-Dimer-001 were subcutaneously injected into the footpad of BALB/c mice respectively. Cryosections of draining popliteal lymph nodes at different time points after vaccination were stained and analyzed by confocal microscopy. Both ATR-AP205-001 vaccine and ATR-Dimer-001 vaccine were drained to the popliteal lymph node within 15 minutes, and resided in the macrophages-rich subcapsular area (Fig. 2A). Fluorescent signal continued to increase in 8 hours and then gradually declined (Fig. 2B). It was noticed that fluorescence of ATR-AP205-001 vaccine almost disappeared in the subcapsular area and significantly accumulated on follicle dendritic cells (FDCs), while ATR-Dimer-001 vaccine showed a random distribution in subcapsule and follicles without co-localization with B cells and FDCs (Fig. 2C). ATR-AP205-001 vaccine accumulated in FDCs area, and kept contact with surrounding B cells for at least 4 days when germinal centers (GCs) start to form, while ATR-Dimer-001 vaccine failed to induce GC formation at the same time point (see Supplementary Fig. S2). It should be mentioned that neither detectable vaccine deposition nor vaccine-capturing cells were observed in spleen at any time point (see Supplementary Fig. S3).

### B cells participated in ATR-AP205-001 vaccine transportation from subcapsule to FDC area

**Figure 3:**
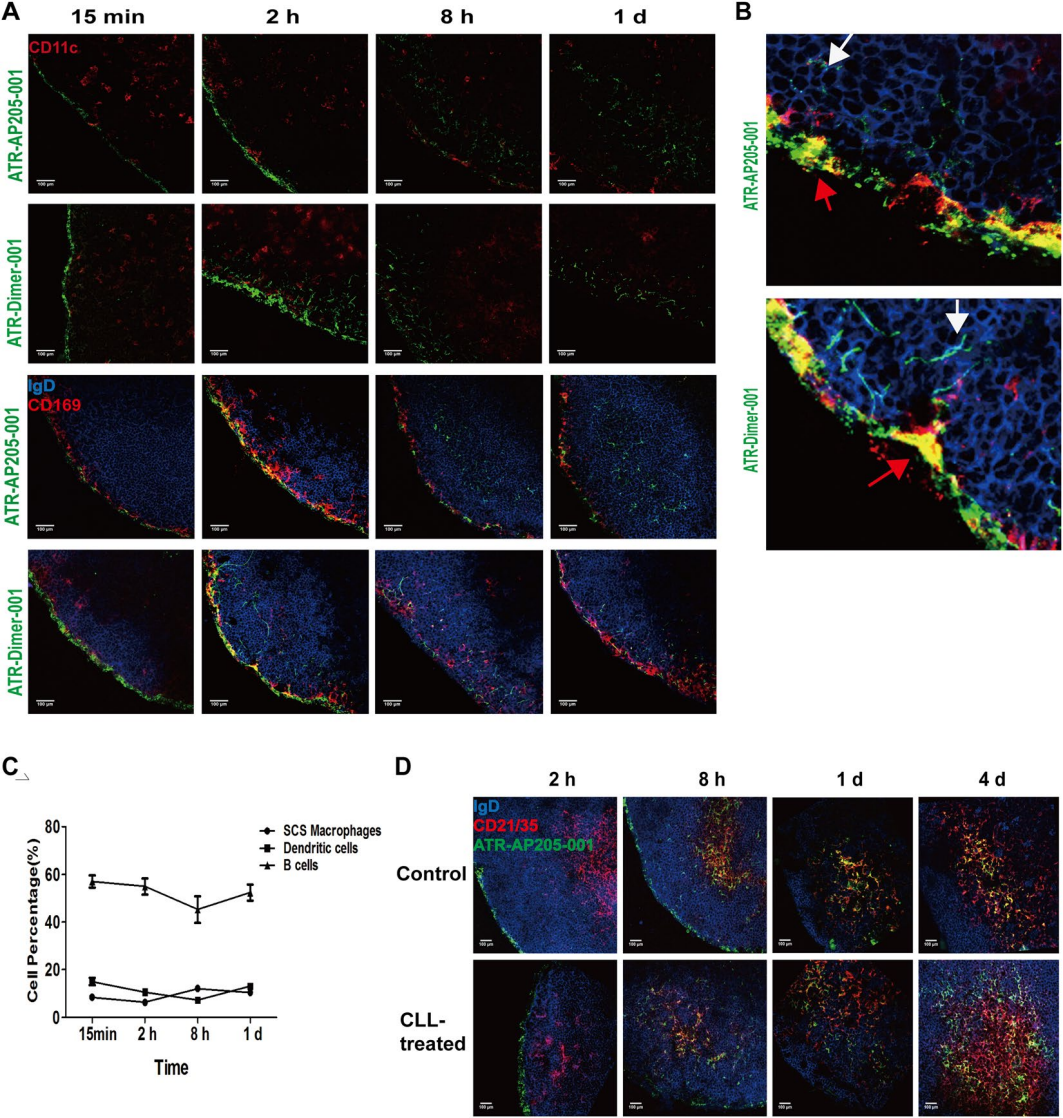
Follicle B cells participated in ATR-AP205-001 transportation. (**A**) Co-localization of ATR -AP205-001 and ATR-Dimer-001 with DCs (CD11c^+^, red), SCS Mφs (CD169^+^, red) and follicle B cells (IgD^+^, blue) in dLNs. (**B**) Higher magnification co-localization images of ATR-AP205-001 and ATR-Dimer-001 with SCS Mφs and follicle B cells after 2 hours vaccination. Red arrows annotate vaccines co-localization with SCS Mφs, white arrows annotate vaccines carried by B cells. (**C**) Proportions of DCs (CD11c^+^), SCS Mφs (CD14^+^CD169^+^), and B cells (CD19^+^) gated on ATR001^+^ population after ATR-AP205-001 immunization. (**D**) Co-localization of ATR-AP205-001 with follicle B cells and FDCs in untreated and CLL-pretreated dLNs. n = 6 per group. Data are expressed as mean ± SEM. Scale bars, 100 μm.

APCs participate in antigens transportation from subcapsule to FDCs. Fluorescent images showed the relative distribution of ATR-AP205-001 vaccine and ATR-Dimer-001 vaccine with DCs, CD169^+^ Mφs and follicle B cells (Fig. 3A). Higher magnification clearly showed that both ATR-AP205-001 vaccine and ATR-Dimer-001 vaccine were caught by CD169^+^ Mφs in SCS area at early time point (Fig. 3B). ATR-AP205-001 signal was scattered deeper in B cell follicle and appeared to be on the surface of B cells for 2 hours after vaccination, while ATR-Dimer-001 vaccine diffused randomly without association with B cells. Flow cytometry showed clearly that follicle B cells accounted for nearly a half of total VLP-based vaccine capturing cells, while SCS Mφs and DCs made up less than 15% respectively (Fig. 3C). The co-localization of ATR-AP205-001 vaccine and ATR-Dimer-001 vaccine with DCs was not directly observed in images and almost all DCs were distributed in T cells zone (Fig. 3A). These results implied the importance of follicle B cells in ATR-AP205-001 vaccine transportation, while DCs may be more important for T cells differentiation. Furthermore, we tried to figure out the effect of SCS Mφs in this process. SCS Mφ clearance with clodronate-encapulated liposomes (CLLs) treatment did not attenuate the accumulation of ATR-AP205-001 vaccine in FDCs area (Fig. 3D). Even though SCS Mφs are just located in subcapsular sinus and so close to follicles, they might perform as a cleaner more than a transporter^16^.

### ATR-AP205-001 vaccine strongly promoted Tfh cells enhancement without biased Th1/Th2/Th17 cells and Treg cells differentiation

**Figure 4:**
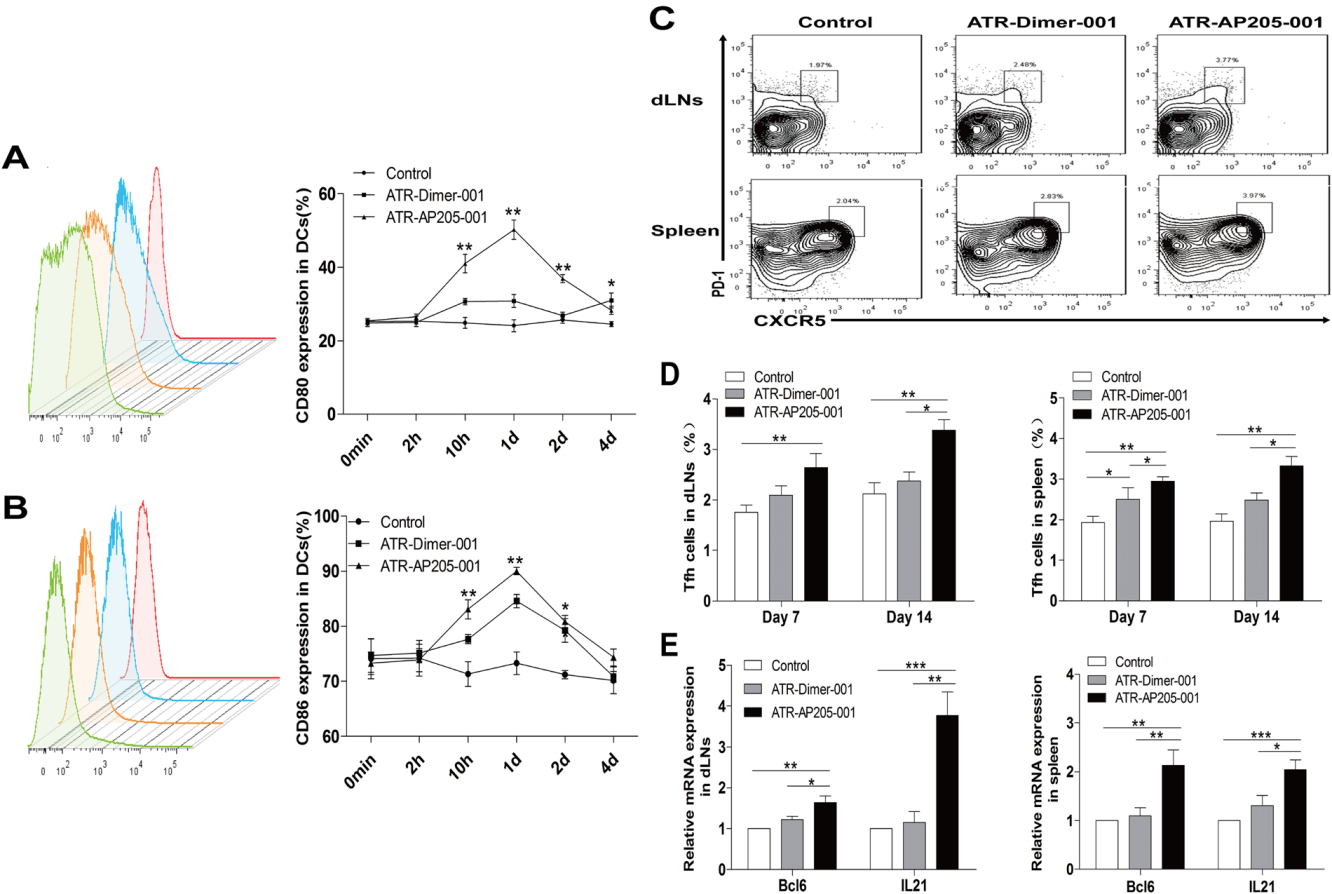
Tfh cells enhancement after ATR-AP205-001 vaccination. The expression of co-stimulatory molecules CD80 (**A**) and CD86 (**B**) on DCs at 0 min, 2 hrs, 10 hrs, 1 day, 2 days and 4 days post vaccination (n = 6 per group). (**C**) PD-1^high^CXCR5^high^ Tfh cells, gated on CD4^+^ T cells. (**D**) Proportion of Tfh cells in dLNs and spleen on days 7 and 14 (n = 7–9 per group). (**E**) Relative mRNA expression of Bcl-6 and IL21 in dLNs and spleen on day 14 (n = 7–9 per group). Data are presented as mean ± SEM. *P < 0.05, **P < 0.01, ***P < 0.001.

ATR-AP205-001 vaccine could rapidly accumulate in FDC area, and continually stimulate B cells response. Meanwhile, ATR-AP205-001 vaccine induced obvious up-regulation of CD80 and CD86 in DCs, far surpassed ATR-Dimer-001 vaccine (Fig. 4A,B). Activation and maturation of DCs contribute to T cells activation and differentiation. Among several T cell subtypes, Tfh cells are the most important helping T cells focusing on promoting GC formation and high affinity B cells screening. Our data showed that ATR-AP205-001 strongly enhanced Tfh cells differentiation, both in the draining lymph nodes and spleen, while ATR-Dimer-001 had little impact on the differentiation of Tfh cells (Fig. 4C,D). Also, RT-PCR showed that the expression level of Tfh-related transcription factor Bcl6 and cytokine IL21 was significantly increased in ATR-AP205-001 vaccination group (Fig. 4E).

**Figure 5:**
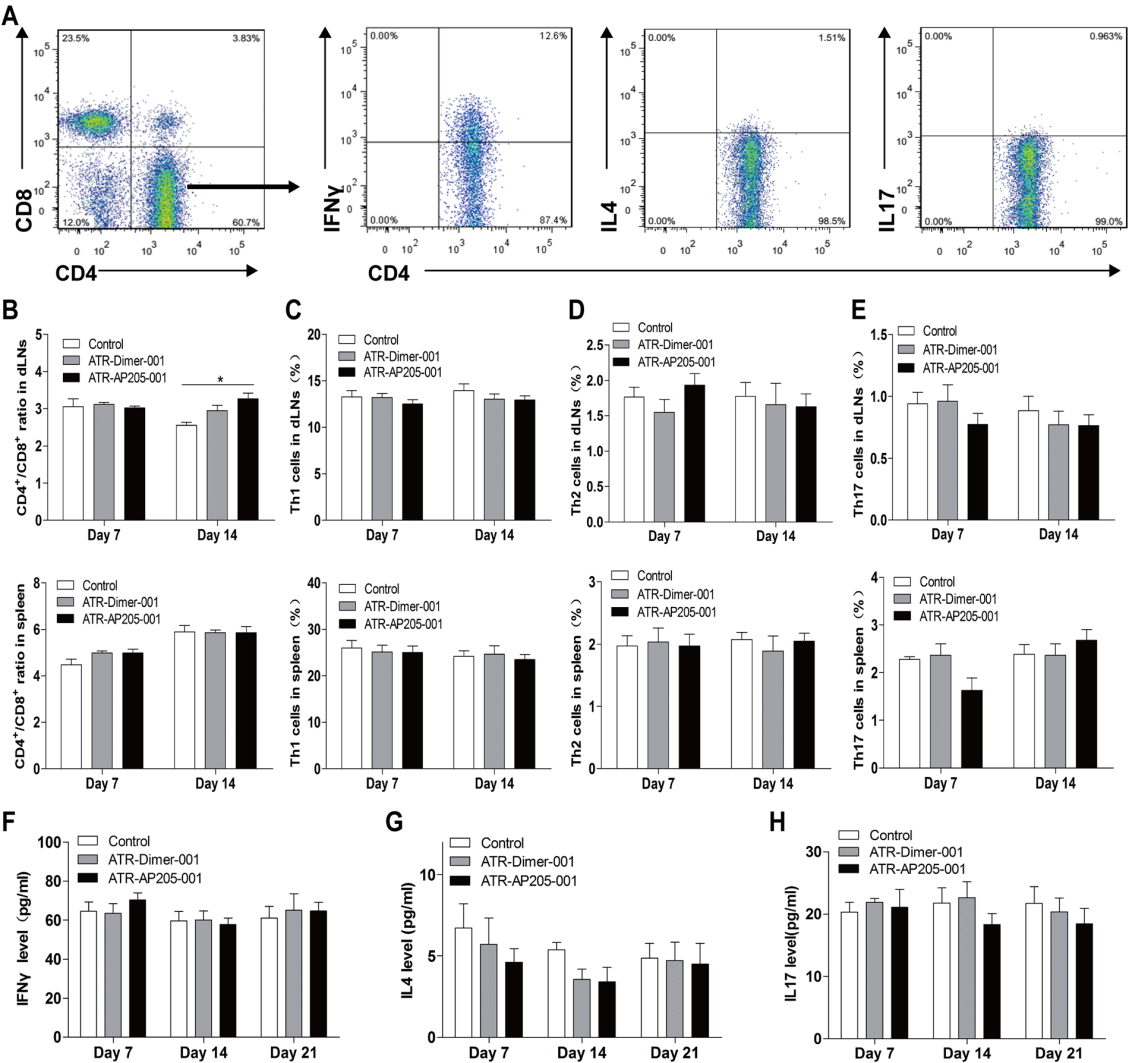
No biased Th1/Th2/Th17 cells differentiation was observed after ATR-AP205-001 vaccination. (**A**) Th1 (IFNγ^+^), Th2 (IL4^+^), and Th17 (IL17^+^) cell gated on CD4^+^ T cells. (**B**) The ratio of CD4^+^ to CD8^+^ T cells and the percentages of Th1 (**C**), Th2 (**D**) and Th17 (**E**) cells in CD4^+^ T cells. Serum concentration of cytokines IFNγ (**F**), IL4 (**G**), and IL17 (**H**) on days 7, 14 and 21. Data are presented as mean ± SEM. n = 6–8 per group. *P < 0.05 vs. control group.

T cell differentiation was further evaluated. Results showed that no significant difference in CD4^+^/CD8^+^ T cell ratio was found between the two vaccination groups on days 7 and 14 after immunization (Fig. 5A,B). And, no obvious Th1/Th2/Th17 biased differentiation was detected in ATR-AP205-001 vaccination group (Fig. 5C–E). Cytokines including IFNγ, IL4, and IL17 in serum also showed no difference between the two vaccination groups (Fig. 5F–H). Furthermore, the mice did not produce enhanced regulatory T cells (Tregs) and suppressive cytokine IL10 after multiple vaccination (see Supplementary Fig. S4

). These results indicated that ATR-AP205-001 enhanced Tfh cells expansion strongly without biased Th1/Th2/Th17 cells and Treg cells differentiation.

### ATR-AP205-001 vaccination enhanced GCs formation, antibody production and long-lasting B cell memory

**Figure 6:**
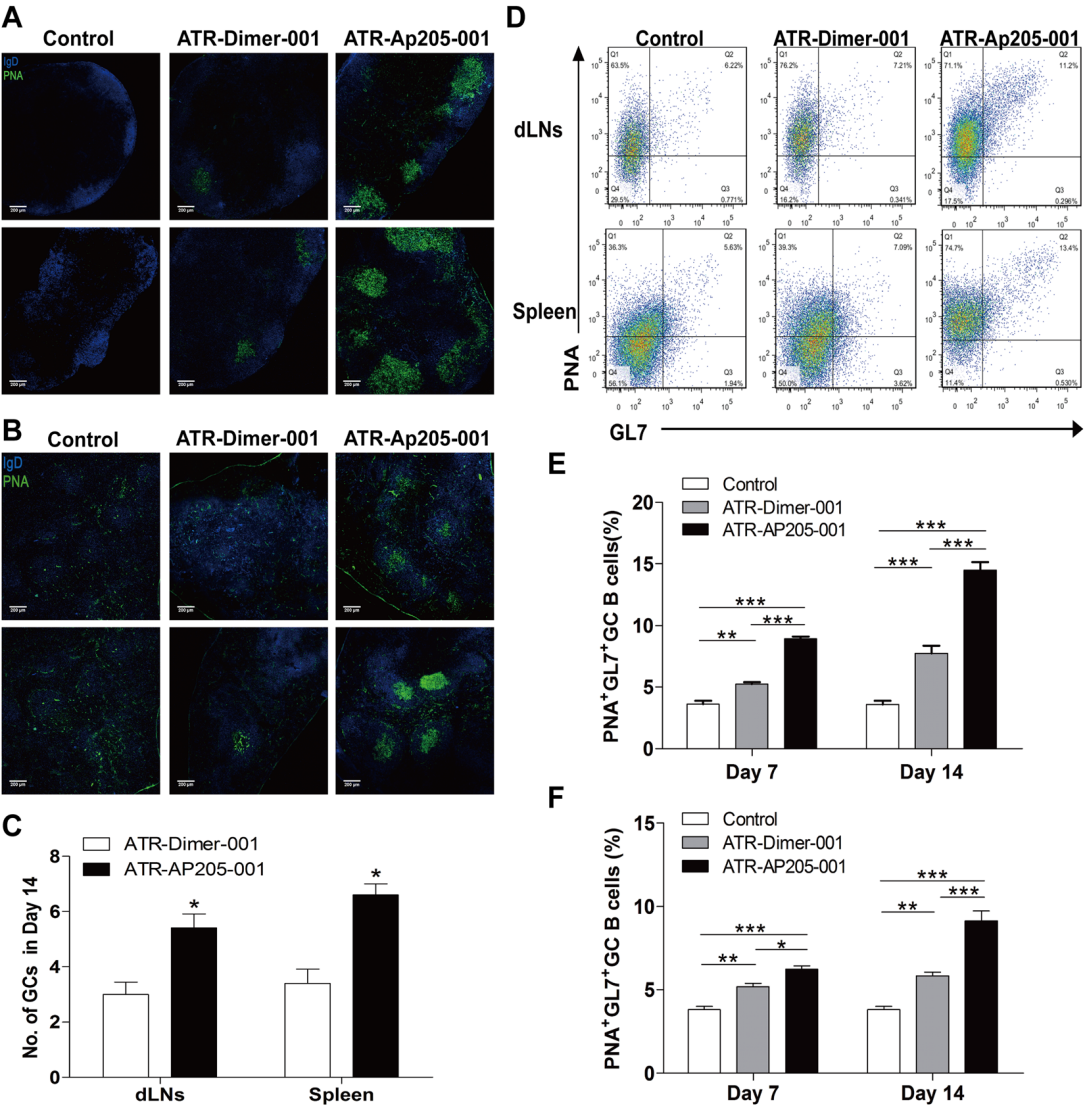
Significant GC formation induced by ATR-AP205-001 vaccination. GC (green) formation in dLNs (**A**) and spleen (**B**). (**C**) Numbers of GCs observed in ATR-AP205-001 and ATR-Dimer-001 group (n = 6 per group). (**D**) GL7^+^PNA^+^ GC B cells gated on CD19^+^ cells. Percentages of GC B cells in dLNs (**E**) and spleen (**F**) (n = 7–9 per group). Data are presented as mean ± SEM. *P < 0.05, **P < 0.01, ***P < 0.001.

As early as 4 days after immunization, activated and proliferating B cells accumulated inside follicles of secondary lymphoid organs, followed by the shaping and development of GCs. ATR-AP205-001 vaccine elicited more enhanced GC response than ATR-Dimer-001 vaccine both in GC size (Fig. 6A,B) and numbers (Fig. 6C). Flow cytometry also showed more activated GC B cells in ATR-AP205-001 vaccination group than in ATR-Dimer-001 vaccination group (Fig. 6D,E,F).

**Figure 7:**
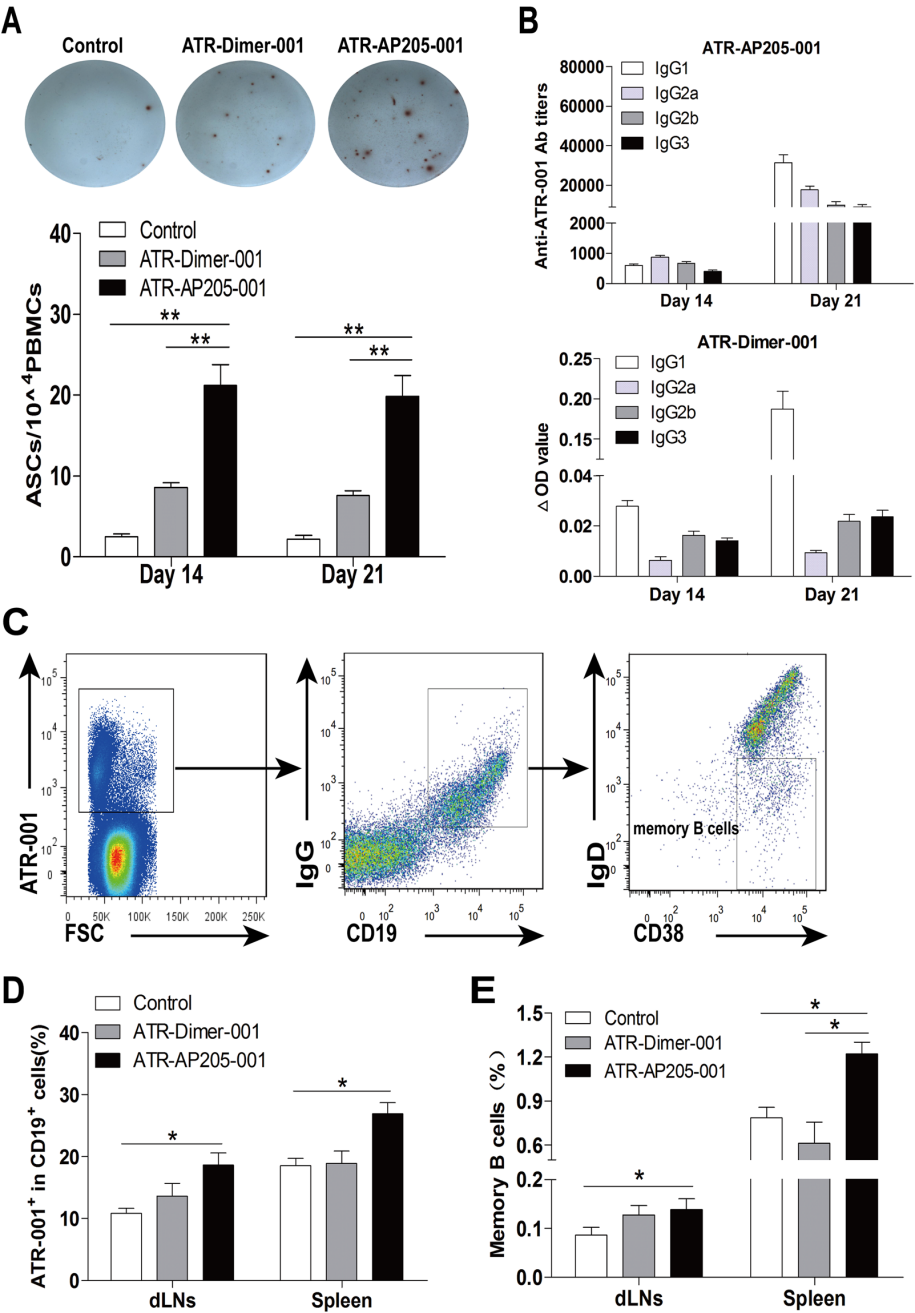
ATR-AP205-001 promoted anti-ATR001 antibody production and long-lasting B cell memory. (**A**) ATR001-specific antibody secreting cells on days 14 and 21. (**B**) IgG isotypes of ATR-AP205-001 and ATR-Dimer-001. (n = 6 per group). (**C**) ATR001-specific memory B cells gated as ATR001^+^ CD19^+^ IgD^−^ IgG^+^ CD38^+^. (**D**) Proportion of ATR001-specific B cells. (**E**) Memory B cells in dLNs and spleen on the 150th days after second immunization (n = 7 per group). Data are presented as mean ± SEM. *P < 0.05 vs. control group.

The expression of AID, Blimp1 and XBP1 that promote plasma cells development was significantly increased in ATR-AP205-001 vaccination group (see Supplementary Fig. S5). In contrast to ATR-Dimer-001 vaccine, ATR-AP205-001 vaccination induced more ATR001-specific antibody secreting cells both after the first and the second immunization (Fig. 7A), the anti-ATR001 antibody titers in ATR-AP205-001 group were also much higher than in ATR-Dimer-001 group (Fig. 1A). Antibody titers against the carrier were also higher in ATR-AP205-001 group(see Supplementary Fig. S6). The subtype of IgG against ATR001 in ATR-AP205-001 vaccine group was IgG1-dominated, similar to ATR-Dimer-001 vaccine (Fig. 7B). Furthermore, ATR-AP205-001 vaccine induced much stronger ATR001-specific B cell memory than ATR-Dimer-001 vaccine in spleen (Fig. 7C–E). All the results showed that ATR-AP205-001 could induce stronger humoral immunity than general protein vaccine.

## Discussion

AT1R-VLP vaccine showed great anti-hypertensive effect and target organs protection in hypertensive, atherosclerosis and nephropathy animal models, without tissue inflammatory lesions as well^4–6^. To accelerate the clinical transformation of AT1R-VLP vaccine, we further explored immune response mechanism of the novel ATR-AP205-001 vaccine. It was found that ATR-AP205-001 vaccine quickly induced potent humoral immunity through collaboration of B cells, follicular dendritic cells and follicular helper T cells.

One of the most prominent phenomenon observed was that ATR-AP205-001 vaccine was freely drained to lymph node rapidly and deposited in FDCs area at a very early time, significantly different from general ATR-Dimer-001 vaccine. Particulate antigens traffic to the draining LN in a size-dependent manner, only small nanoparticles (20–200 nm) could traffic rapidly and freely from skin to lymph node and target LN-resident antigen-presenting cells, facilitating antigens quick and extensive exposure to immune system^17^. ATR-AP205-001 vaccine has an appropriate diameter of 30 nm. Repetitive epitopes presented on the surface were also beneficial for ATR-AP205-001 vaccine to bind to B cells through unspecific BCRs^18,19^. Antigens could be captured and carried in immune complexes (ICs) form^20^. The repetitive structure of particulate antigens could form ICs with natural IgM just after primary exposure^21^. Our results showed that B cells captured ATR-AP205-001 vaccine near the subcapsule of LNs, and were the largest contributor of vaccine capturing. These indicated that ATR-AP205-001 vaccine was also transported to FDCs rapidly through B cells assistance. However, the general protein vaccine ATR-Dimer-001 showed a random distribution in subcapsule and follicles without co-localization with B cells and FDCs. In secondary lymphoid organs, follicle B cells are in very close contact with the network of FDCs in follicles^22^. FDCs continuously present antigens to B cells in the form of ICs and opsonize antigens by Fc and complement receptors respectively^23^. These interactions are important for B cell selection and affinity maturation during the GC response. As we saw, the early and intense GCs formation was observed after ATR-AP205-001 vaccination.

Besides follicle B cells, SCS Mφs also drew our attention. Vaccines from peripheral flow into draining lymph nodes and firstly accumulate in subcapsular sinus where SCS Mφs reside in. Due to their special location, SCS Mφs could perform as the first line to interact with antigens from afferent lymphatic vessels^24^. They participate in phagocytosis and clearance of antigens like virus^24,25^. Meanwhile, several studies identified SCS Mφs in LNs are important in antigens retaining and provide an important site for cognate B cells encountering particulate antigens^20,26,27^. B cells can capture IC from SCS Mφs in a complement receptor dependent way and present IC to FDCs^20^. To estimate the function of SCS Mφs in ATR-AP205-001 vaccine movement, SCS Mφs were depleted by CLLs treatment. Results confirmed that SCS Mφs were dispensable in ATR-AP205-001 vaccine capture and transportation. We supposed that CD169^+^SCS Mφs may have more capacity in controlling ATR-AP205-001 spreading rather than vaccine presenting.

T cell immunity is indispensable part of specific immunity^28^. DCs are the most important and powerful APCs *in vivo*, and the activation and maturation of DCs are key component for vaccine priming T cell immunity^29^. ATR-AP205-001 vaccine markedly facilitated DCs activation and maturation, far surpassing ATR-Dimer-001 vaccine. DCs can display particulate antigens for longer periods of time to enhance production of effective Tfh cells than general antigens vaccine^30^. The strong and specific humoral immunity is the key of ATR-AP205-001 vaccine effect. Tfh cells are the most important helper T cells for humoral immunity. Tfh cells differentiation is driven by expression of the transcriptional repressor B-cell lymphoma-6 (Bcl-6)^31^, which promotes the entry of Tfh cells into follicles and modulates the expression of IL21. IL21 participates in B cells somatic hypermutation and antibody class switching, and is also required for activation of antigen-specific memory B and plasma cells *in vivo* ^32,33^. Nanoparticles as adjuvants were found to selectively induce Tfh cells responses^34^. ATR-AP205-001 vaccine in nanoparticle form enhanced the differentiation of Tfh cells and the production of Bcl-6 and IL21, which triggered stronger B cells response. Such was the case, ATR-AP205-001 vaccine induced intense GCs reaction, a boost of the specific antibody and long-lasting B cell memory, far superior to ATR-Dimer-001 vaccine.

The major limitation of VLP-based therapeutic vaccine compared with other approaches is the safety. Potential excess immunity should be considered carefully. Effective CD4^+^ T cells and secreted cytokines promote proliferation, differentiation and class switching of B cells, while they also contribute to immunopathology in response to antigens^35,36^. CD8^+^ cytotoxic T cells kill targeting cells like infected cells, apoptotic cells and cancer cells, and are potential threat to normal cells^37,38^. Treg cells always act as a regulator to inhibit excess immunity^39^. In this study, to further explore the safety of AT1R-VLP vaccine, T cell differentiation was detected. The results showed that stable CD4^+^/CD8^+^ T cells ratio, Th1/Th2/Th17 cells differentiation and Treg cells numbers in ATR-AP205-001 vaccine were similar to the general protein vaccine ATR-Dimer-001. ATR-AP205-001 also did not induce excess circulatory cytokines as IFNγ, IL4, IL17 and IL10. The analysis of IgG subtypes demonstrated that ATR-AP205-001 vaccine induced an IgG1 dominated humoral immune response as ATR-Dimer-001 vaccine. For antibody-dependent cell-mediated cytotoxicity, still there are several controversies in terms of its onset and regulation. Overall, our previous work demonstrated that AT1R-VLP vaccine showed no obvious inflammation lesions in important organs^4–6^. AngII-VLP hypertension vaccine (CYT006-AngQβ) developed by Cytos Biotechonology showed good safety in clinical trials^40^. These research together with our study﻿ indicated that ATR-AP205-001 vaccine has satisfactory safety. Nevertheless, the potential for antibody-dependent cell-mediated cytotoxicity to be caused by the vaccine will need to be further investigated.

VLP-based vaccines targeting AT1R had been proved to be efficient in preventing multiple diseases like hypertension, atherosclerosis and nephropathy. ATR-AP205-001 vaccine induced quick and strong humoral immunity through the synergic helping of follicular B cells, FDCs and Tfh cells. ATR-AP205-001 vaccine strongly promoted Tfh cells enhancement without biased Th1/Th2/Th17 cells and Treg cells differentiation, which showed the good safety of ATR-AP205-001 vaccine. The findings of this study help us to detail the immune response and safety of VLP-based vaccine. All data support that ATR-AP205-001 may become an efficient and safe intervention for cardiovascular diseases. And, since lots of VLP-based vaccines are being evaluated for treatment of a variety of diseases, this study is also helpful for other VLP-based nanoparticle vaccines.

## Methods

### Animals

Specific Pathogen-Free (SPF grade) male BALB/c mice, weighing 20–25 g, aged 6–8 weeks, were purchased from Hubei Research Center of Laboratory Animal, fed in a specific pathogen-free environment in Laboratory Animal Center, Tongji Medical College, Huazhong University of Science & Technology. All experiments were approved and performed according to the guidelines of the animal care and use ethical committee of Tongji Medical College, Huazhong University of Science and Technology.

### Vaccine preparation

The AP205 VLP prokaryotic expression plasmid was amplified and expressed in *Escherichia coli* (*E. coli*). The procedure of VLP purification and identification followed previous study^2^. In brief, BL21 *E. coli* cultured overnight were lysed completely by ultrasound. The lysate was purified by acidification, sedimentation of saturation ammonium sulfate, hydrophobic interaction chromatography (GE Healthcare), and gel filtration chromatography (GE Healthcare). Dimer was produced through depolymerization of purified VLP, sedimentation, resolvation and purification by hydrophobic interaction chromatography. N-Ethylmaleimide (NEM, Sigma Aldrich) was used to block sulfhydryl group in dimer protein to avoid useless thioether bonding with crosslinkers. Single ATR001 peptide (A-F-H-Y-E-S-Q) and FITC conjugated-ATR001 peptide were customized from GL biochem of Shanghai. Analyzed by high performance liquid chromatography and mass spectrometry, the purity of peptides reached 95%. Peptides were covalently conjugated to VLP and dimer respectively in a mass ratio of 1:3 through Sulfo-SMCC crosslinker (Pierce Biotechnology) to produce ATR-AP205-001 vaccine and ATR-Dimer-001 vaccine. Conjugation efficiency of the productive vaccines was identified by SDS-PAGE (see Supplementary Fig. S7). Coomassie Brilliant Blue protein assay kit was used to detect vaccine concentration.

### Animal experiment

To observe bio-distribution and deposition of vaccine in peripheral lymphoid organs, FITC-conjugated vaccine was injected into the footpad and nape back of mice subcutaneously with a dosage 10 μg and 100 μg respectively. For depletion of lymph node macrophages, footpad of BALB/c mice was injected with 10 μl CLLs or empty liposomes (Science Park 408, Building VI, 1098 XH Amsterdam, The Netherlands) on days -5 and -1 before vaccination. For specific immunity study, BALB/c mice were immunized with ATR-AP205-001 vaccine and ATR-Dimer-001 vaccine on days 0 and 14, with a dosage of 100 μg per mice. For long-term inflammation observation, BALB/c Mice were immunized 5 times with a dosing interval of 14 days. For the study of the antihypertensive effect, mice were anesthetized with 3.5% chloral hydrate on day 21. Then Ang II-infused osmotic pumps (1.4 mg/kg/day) were planted subcutaneously in the back of the mice for another 14 days. Mice treated with valsartan (10 mg/kg/day) by intragastric administration were set﻿ as﻿ a positive control. Blood pressure was measured per 5 days by tail-cuff method (softron BP98A, Japan). Mice were sacrificed on day 42.

### Reagents and antibodies

Fitc-conjugated anti-CD4 antibody, PE-conjugated anti-IFNγ antibody, APC-conjugated anti-IL4 antibody, PE/Cy7-conjugated anti-IL17 antibody, and Percp/Cy5.5-conjugated anti-CD8 antibody were purchased from ebioscience. Biotin-conjugated PNA was from Vector lab. APC-conjugated anti-GL7 antibody, PE-conjugated anti-PD-1antibody, APC-conjugated anti-CXCR5 antibody, PE-conjugated anti-CD169 antibody, APC-conjugated anti-CD11c antibody, PE/Cy7-conjugated anti-CD14 antibody, Percp/cy5.5-conjugated anti-CD19 antibody, pacific blue-conjugated anti-IgD antibody, PE-conjugated anti-CD21/35 antibody, PE-conjugated anti-CD19 antibody, Alex Fluor@488-conjugated streptavidin, PE-conjugated streptavidin, Fitc-conjugated anti-CD80 antibody, and PE/Cy7-conjugated anti-CD86 antibody were obtained from biolegend. Mouse IL4, IL17, IL10, and IFNγ ELISA kit were bought from Neobioscience. Lymphocytes Separation Medium was purchased from MP Biomedicals, LLC. Micro-osmotic pump for 14 days was from DURECT Corporation (Cupertino, CA, USA). MultiScreen@HTS96-well plates were obtained from EMD Millipore Corporation (Billeria, MA, USA).

### Immunofluorescence

lymph nodes and spleen were covered by OCT and made into frozen section of 7μm thickness. The sections were dried in room temperature for 20 minutes, fixed in precooling acetone for 10 minutes, washed with PBS for 3 times, then blocked with 10% (v/v) goat serum and 2% (w/v) bovine serum albumin for 30 minutes. Fluorescent antibodies were added and incubated at 4 °C overnight. Residual antibody was washed away the next day. Sections were dried and sealed with anti-quenching agent. We used a laser confocal microscope for imaging.

### Flow Cytometry

Lymph nodes and spleen were separated and grinded to cell suspension. Density gradient centrifugation was used to isolate mononuclear cells. Appropriate concentration of antibodies was added to 100 μl cell suspension at 4 °C for 30 minutes. Cells were then washed twice with PBS and resuspended with cell staining buffer (BD Pharmingen). BD LSRII flow cytometer system (BD Biosciences, USA) was used to test the cell samples. The data was analyzed by Diva and FlowJo 7.6.1.

### Enzyme Linked Immunosorbent Assay (ELISA)

For serum cytokine testing, serum was diluted as a ratio of 1:2. For antibody testing, serum samples were diluted as a gradient ratio of 1:500, 1:1000, 1:5000, 1:10000, 1:20000, 1:40000 and 1:80000. Diluted samples were added to 96 well plate pre-coated with ATR001 peptide, and incubated at 37 °C for two hours. After washed 5 times with PBS-Tween 20 (0.05%, v/v) solution, the plate was covered with Biotin-labeled antibody and incubated for 1 hour at 37 °C. HRP-labeled streptavidin was added to bind with biotin-labeled antibody for another 1 hour, and TMB Chromogenic Solution was used to react with HRP for color reaction. Microplate-reader for ELISA was used to detect half-maximal OD values of the sample wells.

### Enzyme Linked Immunospot (ELISPOT) Assay

HTS96-well plates were coated with ATR001 peptide (2 μg/well) over night, washed with sterile PBS, and blocked by RPMI1640 culture medium containing 10% (v/v) FBS for 2 hours. Thigh bone was isolated from sacrificed mice and used to provide bone marrow monocytes. These monocytes were suspended in culture medium containing 10% FBS, and added to the coated plate in a concentration of 1 × 10^4^ cells/well. After incubation in 37 °C, 5% CO2 incubator for 24 hours, cells in the plate were lysed by pure water, and then washed five times with PBS-Tween 20(0.05%) solution. HRP-labeled goat anti-mouse IgG antibody was added to the plate and incubated for 1 hour at room temperature. AEC solution (Boster bio-engineering, Wuhan, China) was used to color the binding site of HRP-labeled antibody. The results were observed and coloring spots were calculated under dissecting microscope.

### RNA isolation and Reverse Transcription Polymerase Chain Reaction (RT-PCR)

Tissue RNA was isolated by RNAiso Plus (Takara). RNA was reversely transcribed in a 20 μl-reaction system containing Primescript RT Master Mix for RT-PCR (Takara). The obtained cDNA was then mixed with PCR primers in SYBR Premix Ex TaqTM (Takara) and amplified in Bio-Rad detection system as following procedure: an initial denaturation cycle lasting 30 seconds (s) at 95 °C, followed by 40 cycles of amplification each comprising denaturation for 3 s at 95 °C, and annealing for 30 s at 60 °C. Primer sequences were shown in Table 1.

**Table 1:** Primer Sequences of molecules.

Molecule	Primer Sequence (5′ to 3′)
β-actin	Forward CTGAGAGGGAAATCGTGCGT Reverse CCACAGGATTCCATACCCAAGA
IL21	Forward TCAATGCAGCACAGGCTAAG Reverse GGTTCAAGACTGCTGTGCAA
Bcl6	Forward ATGAGATTGCCCTGCATTTC Reverse GCAGGACTCTGTGGGTGAGT
XBP-1	Forward GGAGCAGCAAGTGGTGGATTT Reverse GTGTCCATTCCCAAGCGTGTT
AID	Forward AGGGACGGCATGAGACCTAC Reverse TCCATCTCAGAAACTCAGCCAC
Blimp-1	Forward CATGGAGGACGCTGATATGAC Reverse ATGCCTCGGCTTGAACAGAAG

### Statistical Analysis

All the data were presented as mean ± SEM. Statistical difference was analyzed by student’s t-test between two groups, and one-way analysis of variance followed by Bonferroni test was used for multiple groups (GraphPad prism 5). p < 0.05 was accepted as significant.

### Data Availability

All data generated or analysed during this study are included in this published article (and its Supplementary Information files).

## Electronic supplementary material

Supplementary information
