# Supplementary material for: Immune Response of A Novel ATR-AP205-001 Conjugate Anti-hypertensive Vaccine
Source: Sci Rep. 2017 Oct 3;7:12580. doi: 10.1038/s41598-017-12996-y (PMC5626684; doi:10.1038/s41598-017-12996-y)
Supplement: Supplementary file 1 — Supplementary information [file 41598_2017_12996_MOESM1_ESM.pdf]

Supplementary Information

Immune Response of A Novel ATR-AP205-001 Conjugate Anti-hypertensive Vaccine

Xiajun Hu[1,2,3,*], Yihuan Deng[1,2,3,*], Xiao Chen[1,2,3,*], Yanzhao Zhou[1,2,3], Hongrong Zhang[1,2,3], Hailang Wu[1,2,3], Shijun Yang[1,2,3], Fen Chen[1,2,3], Zihua Zhou[1,2,3], Min Wang[1,2,3], Zhihua Qiu[1,2,3], Yuhua Liao[1,2,3]

[1]Department of Cardiology, Union Hospital, Tongji Medical College, Huazhong University of Science and Technology, Wuhan 430022, China. [2]Institute of Cardiology, Union Hospital, Tongji Medical College, Huazhong University of Science and Technology, Wuhan 430022, China. [3]Key Lab of Molecular Biological Targeted Therapies of the Ministry of Education, Union Hospital, Tongji Medical College, Huazhong University of Science and Technology, Wuhan 430022, China.

[*]These authors contributed equally to this work.

Correspondence should be addressed to Y.L. (liaoyh27@163.com) and Z.Q. (qiu_zhihua512@163.com)

Supplementary Figure S1

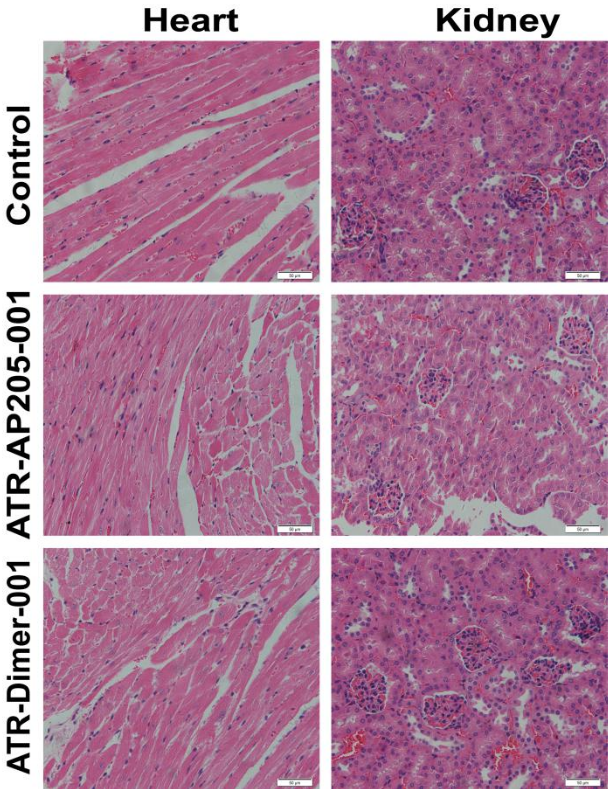

Figure S1. No immunologic damages were observed after ATR-AP205-01 or ATR-Dimer-001 vaccination. H&E staining of heart and kidney from mice after 5 times vaccination (n=6 per group). Scale bars, 50μm.

Supplementary Figure S2

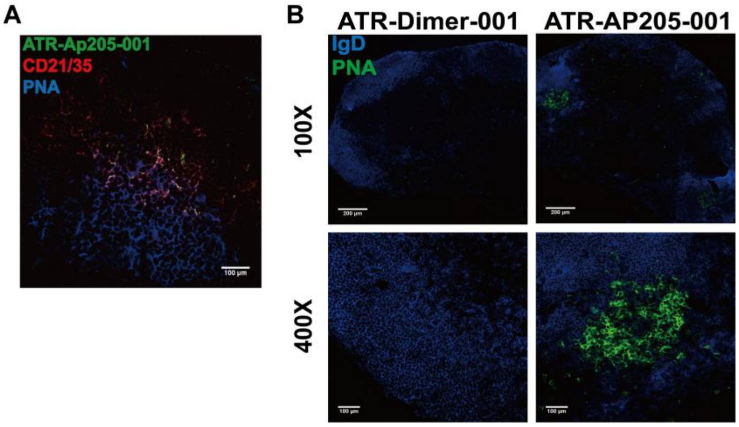

Figure S2. Early GC formation 4 days after ATR-AP205-001 vaccination. (A) Colocalization of GC (blue) and FDCs (red) presenting ATR-AP205-001 (green). Scale bars, 200µm. (B) GC staining 4 days after ATR-Dimer-001 or ATR-AP205-001 vaccination.100X, Scale bars, 200µm. 400X, Scale bars, 100µm.

Supplementary Figure S3

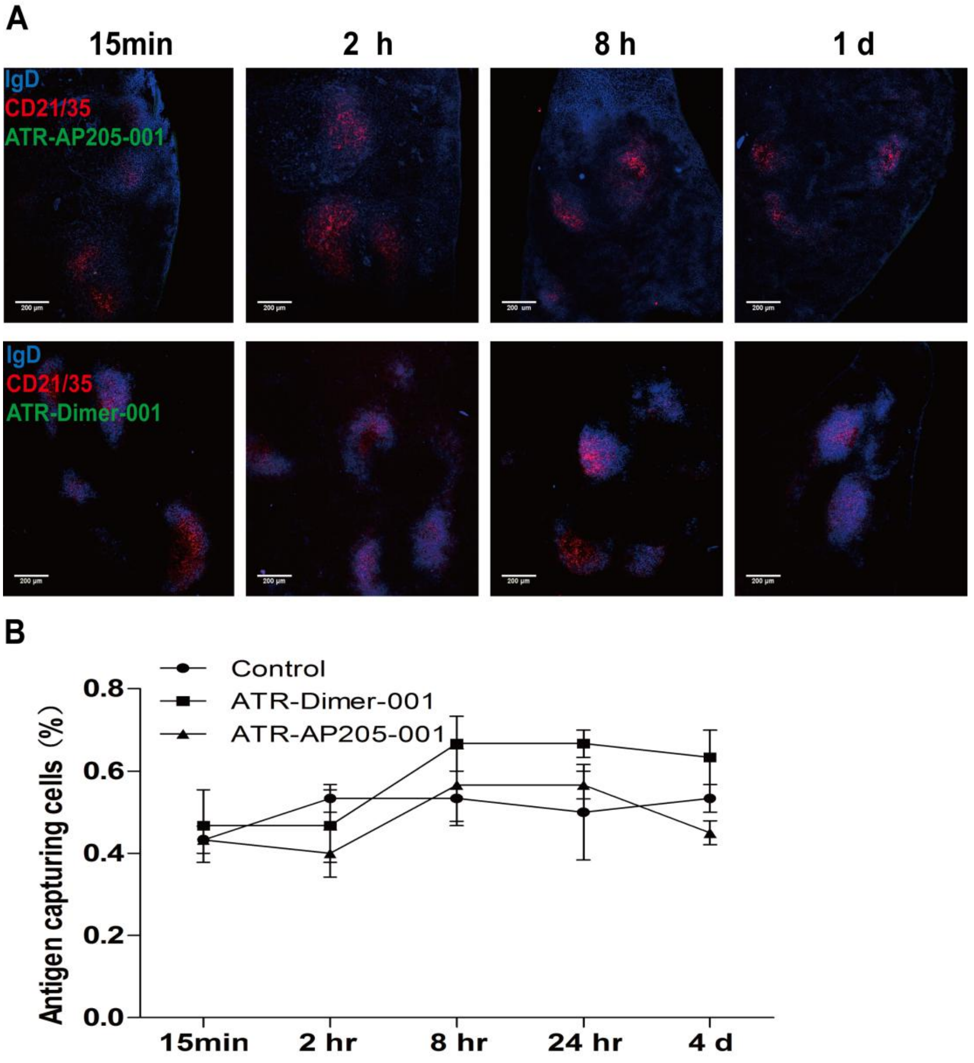

Figure S3. No fluorescent ATR-AP205-001 or ATR-Dimer-001 were observed in spleen. (A) Fluorescent images of spleen after immunization (n=6 per group). Scale bars,200μm. (B) Percentages of Antigen-capturing cells in total cells determined by flow cytometry (n=6 per group). Data are presented as mean ± SEM.

Supplementary Figure S4

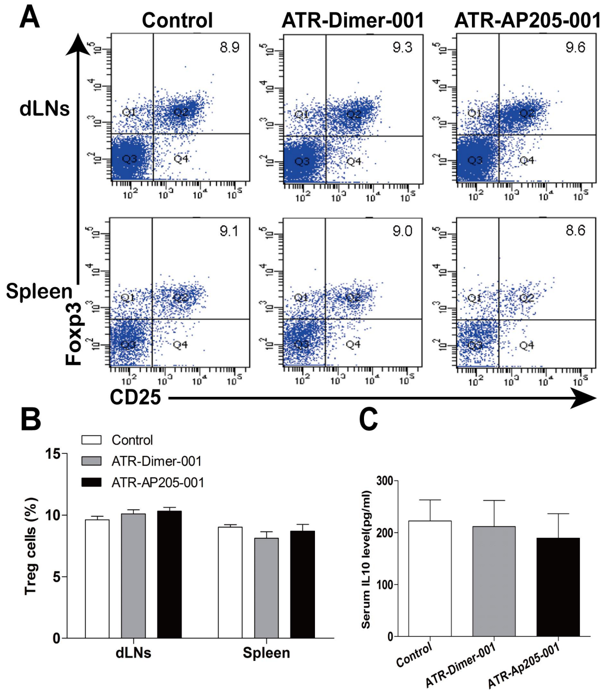

Figure S4. No Treg cells differentiation was observed after multiple vaccination. (A) Treg ($CD25^{+}Foxp3^{+}$) gated on $CD4^{+}$ T cells. (B) Percentages of Treg cells in $CD4^{+}$ T cells after 5 times immunization (n=7 per group). (C) Serum IL10 concentration (n=7 per group). Data are presented as mean ± SEM.

Supplementary Figure S5

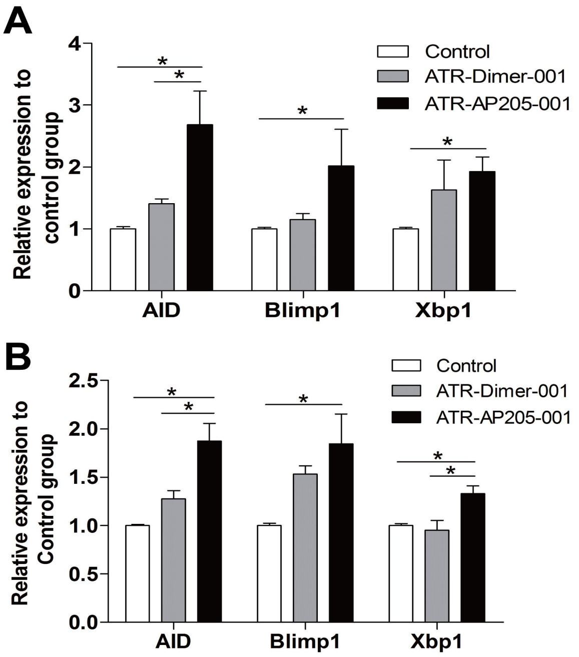

Figure S5. Relative mRNA expression of AID, Blimp1 and Xbp1 in dLNs and spleen on day 14 (n=7 per group).Data are presented as mean ± SEM. *P < 0.05.

Supplementary Figure S6

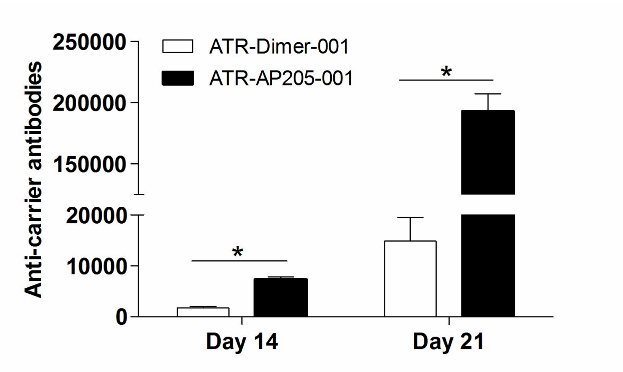

Figure S6. Antibody titers against the carriers in ATR-Dimer-001 or ATR-AP205-001 group. Data are presented as mean ± SEM. *P < 0.05 vs ATR-Dimer-001 group.

Supplementary Figure S7

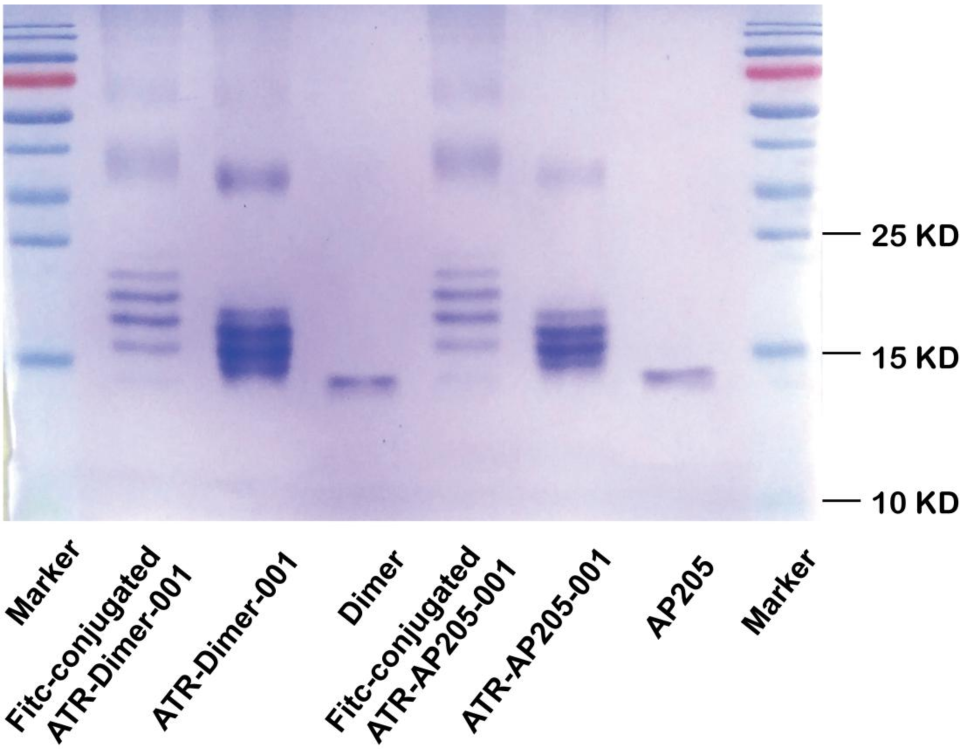

Figure S7. SDS-PAGE analysis of carriers and vaccines by eluting gel stained with coomassie brilliant blue.
